# Analysis of Leafy Vegetable Nitrate Using a Modified Spectrometric Method

**DOI:** 10.1155/2018/6285867

**Published:** 2018-08-05

**Authors:** Tzu-Hsien Yu, Shuo-Ping Hsieh, Chien-Ming Su, Feng-Jung Huang, Chien-Che Hung, Lih-Ming Yiin

**Affiliations:** ^1^Department of Public Health, Tzu Chi University, Hualien, Taiwan; ^2^Institute of Medical Sciences, Tzu Chi University, Hualien, Taiwan

## Abstract

A quick and cost-effective method to analyze leafy vegetable nitrate on spectrometry was developed and compared with a standard method using high performance liquid chromatography (HPLC). This method was designed to use ion-exchange solid phase extraction (SPE) cartridges in reducing interference from organic matrices to meet the criterion of an existing method for analyzing nitrate in wastewater. Nine vegetables (bok coy, cabbage, celery, Chinese cabbage, Chinese kale, lettuce, mustard green, pak choi, and water spinach) were selected for the method testing with three replicates being conducted for each vegetable. The nitrate contents ranged from 800 to 4,300 *μ*g/g, with bok coy, celery, and pak choi being the highest. Data derived from spectrometry and HPLC were close to each other with most relative errors being within ±10% and were highly correlated with an R square value of 0.969. Stability testing and spike analysis resulted in a mean coefficient of variation lower than 6% and a mean recovery rate of 83.7%, suggesting reliability of the method. In addition, both the cost and time consumption for using this method were lower than the standard method using HPLC or ion chromatography, making this spectrometric method a good alternative for analysis of leafy vegetable nitrate.

## 1. Introduction

Nitrate, ubiquitously present in the environment as one of the components in the nitrogen cycle, is an essential nutrient to the growth of vegetables. It is common that vegetables contain levels of nitrate, especially those with application of nitrogenous fertilizers. Generally speaking, the levels of nitrate in vegetables are not toxic to most people but are of special concern to newborns. The toxicity of nitrate is mainly attributable to its reduction to nitrite after ingestion. Nitrite, inducing methemoglobin that interferes with oxygen carrying capacity of hemoglobin, causes methemoglobinemia to which infants under 3 months old are particularly susceptible [1, 2]. The American Academy of Pediatrics suggests avoiding home-prepared infant foods from vegetables until children are older [3].

In contrast to toxicity, nitrate has been found to be beneficial to human health. Dietary inorganic nitrate, especially derived from vegetables, serves as a substrate for endogenous nitric oxide (NO), which has been confirmed to possess functions of regulating cardiovascular homeostasis, attenuating oxidative stress and reducing the oxygen cost of moderate-intensity exercise [4–6]. Several studies have shown effects of nitrate on reducing blood pressure [7–9], lowering the risk of stroke [10], and preventing Barrett's oesophagus [11]. Although there are inconsistent results for cardiovascular/exercise benefit of nitrate, it is suggested that vegetable nitrate intake, rather than nitrate supplementation, may provide better benefits to health [12].

Analysis of nitrate in vegetables is certainly necessary to assess exposure to nitrate for adverse or beneficial effects. A quick method is to use a strip test kit, which immediately shows the concentration ranges or levels but is unable to indicate the exact values. To obtain the accurate concentrations, vegetable samples have to be homogenized, processed, and analyzed on instrumentation, including spectrometry [13], ion chromatography (IC) [14–16], high performance liquid chromatography (HPLC) [17–20], and capillary electrophoresis [21–23]. These methods are good at analyzing nitrate and nitrite in foodstuff simultaneously with accuracy and precision, but the analyses either involve use of toxic substances or are relatively costly. Because of nearly no or little nitrite detected from leafy vegetables [15, 17], a method that focuses on vegetable nitrate with similar accuracy and precision plus cost effectiveness would be an excellent replacement.

We developed a quick and cost-effective method for analyzing leafy vegetable nitrate by modifying a spectrometric method used for analysis of nitrate in wastewater (NIEA W419.51A) [24]. The method for wastewater nitrate analysis could not be applied to food analysis because of interference from organic matrices. In this paper, we attempted to reduce the matrix interference utilizing ion-exchange solid phase extraction (SPE) cartridges and successfully met the criterion of method availability. Data derived from analysis using this method were close to that using a standard HPLC method, indicating that this modified method could be a good alternative for analysis of nitrate in leafy vegetables.

## 2. Materials and Methods

### 2.1. Leafy Vegetables

Fresh vegetables, including bok coy, cabbage, celery, Chinese cabbage, Chinese kale, lettuce, mustard green, pak choi, and water spinach, were commonly available and purchased from local markets in Hualien, Taiwan, from April 2016 to July 2017.

### 2.2. Chemicals

Potassium nitrate (≥ 99%, Certified ACS grade), sodium chloride (≥ 99%, Certified ACS grade), and phosphoric acid (85%, Certified ACS grade) were purchased from J.T. Baker® (Thermo Fisher Scientific, Waltham, MA USA); methyl alcohol (99.8%, ACS Reagent grade) and octylamine (99%, Reagent grade) were obtained from Macron™ Chemicals (Capitol Scientific, Austin, TX USA) and Sigma-Aldrich (St. Louis, MO USA), respectively.

### 2.3. Materials and Equipment

Deionized (DI) water used in the process was generated from Purist® UV Ultrapure Water system (RephiLe Bioscience, Ltd., Boston, MA, USA), and a centrifuge (Heraeus™ Megafuge™ 16R, Thermo Fisher Scientific, Waltham, MA USA) was used to process the homogenized samples. For the spectrometric analysis, ion-exchange SPE cartridges (Oasis MAX 6 cc Vac Cartridge, 150 mg, 30 *μ*m, Waters Corp., Milford, MA, USA) were first used to remove matrix interference from sample solutions, which were later analyzed on a spectrometer (NanoDrop™ 2000c, Thermo Fisher Scientific, Waltham, MA USA). For HPLC assay, each sample solution was filtered with a syringe filter (Nylon Membrane, 25 mm × 0.45 *μ*m, Pall Corp., Port Washington, NY USA) and then analyzed on an HPLC system (Agilent Technologies 1200 Series, Santa Clara, CA USA) equipped with a C18 column (Zorbax® 5 *μ*m SB-C18, 250 × 4.6 mm, Agilent Technologies, Santa Clara, CA USA).

### 2.4. Sample Processing and Analysis

Vegetables were washed with tap water and then DI-water prior to process. Twenty grams of each vegetable was homogenized with 100 mL of DI-water and boiled for 15 minutes. After cool-off, the mixture was transferred to a centrifuge tube for 10-minute centrifugation at 3000 rpm. Supernatant was selected and divided into two portions for analyses on spectrometry and HPLC. Three replicates of each vegetable coming from the same bunch were performed for quality control.

For analysis on spectrometry, the supernatant was diluted to one tenth and 1 mL of the solution was added to an ion-exchange SPE cartridge, which was preconditioned with 2 mL of DI-water. After the solution dripped off, 10 mL of DI-water was added to wash out water-soluble organics, followed by a solution of 0.5 M sodium chloride to elute the component of nitrate. Five milliliters (5 mL) of the eluate was collected for analysis on spectrometry, which made the process on SPE cartridge a dilution of 5 times (1 mL in and 5 mL out).

Nitrate in aqueous solution results in an absorbance peak at wavelength of 220 nm and no absorbance at 275 nm. To meet the criterion of the spectrometric method (NIEA W419.51A), two folds of the absorbance at 275 nm have to be smaller than 10% of the absorbance at 220 nm; otherwise, the matrix interference is too big to be omitted. After each sample met the criterion, the net absorbance (Abs) for nitrate was calculated using the following equation and converted into the nitrate concentration via a calibration curve, set by a series of standard solutions with concentrations from 0 to 25 *μ*g/mL. The standard solutions including a blank were also prepared in 0.5 M sodium chloride solution.

(1) Net Abs for NO3−=Abs@220  nm−2×Abs@275  nm

The derived concentration was multiplied by the total weight of homogenized sample (120 g) and dilution factors (i.e., 5, 10) and divided by the weight of vegetable (20 g) to become the vegetable nitrate content (*μ*g/g). The limits of detection and quantification were approximately 0.1 and 0.33 *μ*g/mL, respectively.

**Figure 1:**
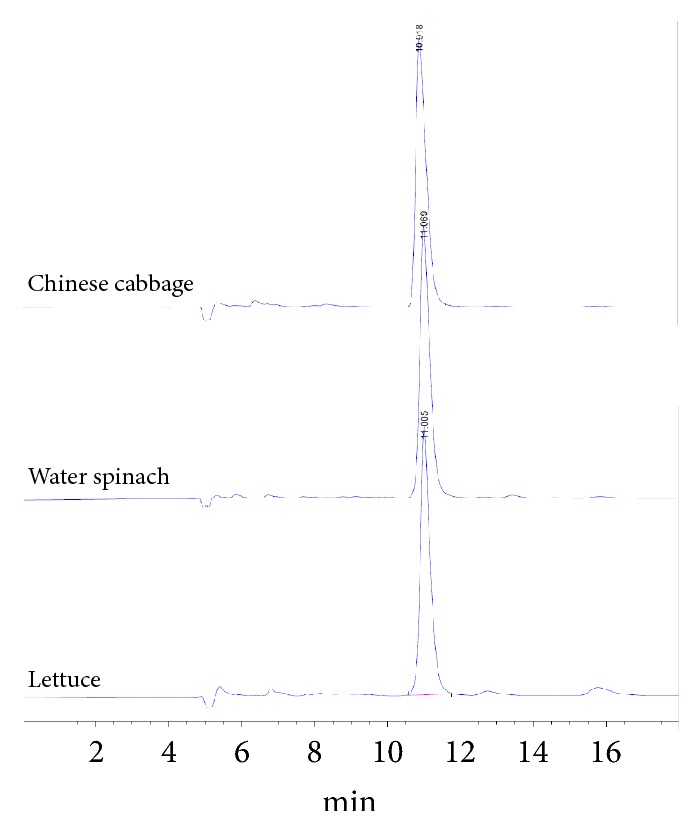
HPLC chromatograms of selected vegetables.

For HPLC analysis, the supernatant was diluted to one fiftieth to match the dilution for analysis on spectrometry, and 1 mL of the solution was filtered with a 0.45 *μ*m syringe filter before analysis on instrumentation. We applied and modified the HPLC method described in the work of Chou et al.'s [17], using a 0.01 M octylammonium orthophosphate solution in 30% (v/v) methanol (pH 7.0) as mobile phase with a flow rate of 0.5 mL/min. The peak of nitrate appeared around 11.0 min with the wavelength for detection set at 213 nm (Figure 1). The vegetable nitrate content (*μ*g/g) was calculated in a similar way to that used in the spectrometric method.

### 2.5. Stability Test and Spike Analysis

Quality check, such as stability test and spike analysis, was conducted for all 9 vegetables (separate procedures from regular analyses). The purpose of stability test was to determine whether the processed vegetable samples were as good as fresh within a week and also to check the precision of this spectrometric method. The vegetable samples were repeatedly analyzed on spectrometry on days 1, 2, 3, 5, and 7 and stored at 4°C in a fridge between analyses.

For spike analysis, a homogenized sample was equally divided into two portions, one of which was processed with 100-mL water as described in Section 2.4 and the other was spiked with an equivalent standard nitrate solution (500 *μ*g/mL). The regular and spike samples were centrifuged, processed with the SPE cartridges, and analyzed on spectrometry following the abovementioned procedure to calculate the recovery rates.

### 2.6. Statistics

Descriptive statistics (e.g., mean, standard deviation) was conducted for all results, and paired t-test was used to compare the results derived from the spectrometric and HPLC methods. A linear regression relationship was established to facilitate data conversion between the two analyses. The statistics was performed using MS Excel 2010 or SPSS 19 on Windows 10 system.

## 3. Results and Discussion

### 3.1. Analytic Results of Vegetables 

All vegetable samples were diluted to one fiftieth prior to analysis on spectrometry or HPLC. After the ion-exchange SPE treatment, all sample solutions analyzed on spectrometry successfully met the criterion of availability, which was twofold of the absorbance at 275 nm smaller than 10% of the absorbance at 220 nm. Concentrations of the diluted solutions lay between 1 and 20 *μ*g/mL and were converted to the nitrate contents of vegetables, which ranged from 800 to 4,300 *μ*g/g with bok coy, celery, and pak choi being the highest (Table 1). The concentration range of the vegetables analyzed in this study was in agreement of results reported by previous studies [15, 17, 25], confirming the nitrate contents in these regularly consumed vegetables. Although replicates were conducted with the same bunches of vegetables, within-the-vegetable variances were different. Cabbage and mustard green had consistent results with very small standard deviations (< 50 *μ*g/g), whereas celery yielded large ones among replicates (740 and 940 *μ*g/g for spectrometry and HPLC, respectively). Differences among replicates may have resulted from uneven distributions of nitrate in the same vegetables or simply from different individual vegetables. For example, a bunch of celery for sale in a market may consist of three or more individuals, which are likely to contain various contents of nitrate due to many factors, such as application of fertilizers and exposure to sunlight, as suggested by Chen et al. [15].

### 3.2. Comparison between Spectrometry and HPLC

Comparisons between results derived from spectrometric and HPLC analyses are also listed in Table 1. Most of the relative errors were lower than 10%, indicating that data generated from the spectrometric method was close to that from HPLC. Despite the closeness, the difference between the two methods was statistically significant (P = 0.013, not shown in table), showing that 7 of the 9 vegetables yielded slightly higher results from spectrometry than from HPLC. It is suggested that, other than random errors, there could have been little interference in the samples that happened to have absorbance at 220 nm as did nitrate. Because the ion-exchange SPE cartridges were functional to separate organic matrices from ions, the interference must have been other anions. One possibility was nitrite, which also had absorbance at 220 nm and was reported in near zero or little quantities present in vegetables [15, 17]. Albeit the little interference, this spectrometric method is nearly as good as the HPLC method; the slightly higher values, if necessary, could be corrected using an empirical formula derived from a linear regression analysis, which is listed in the next section.

### 3.3. Regression Analysis

**Figure 2:**
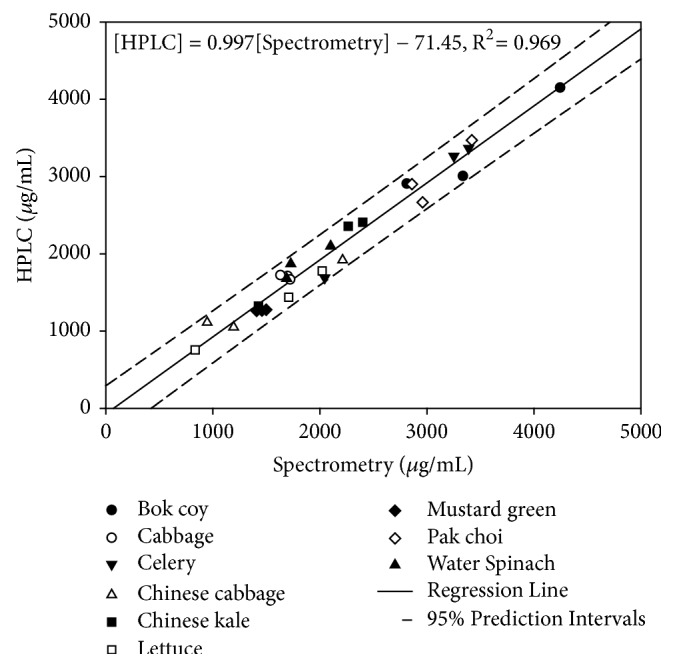
Linear regression for pairs of vegetable nitrate data derived from spectrometry and HPLC. The curved lines represent 95% confidence limits on an individual predicted HPLC value for a given value of spectrometry.

Results of three replicates of the 9 vegetables are plotted in Figure 2. A linear regression relationship between the two methods was established with the relevant statistical parameters shown as follows and in Table 2:

(2) HPLC=0.997Spectrometry−71.45,R2=0.969

The slope was approximately 1.0, indicating the consistency between the results derived from the two methods. All the 27 points, representing 9 various vegetables, were located within 95% confidence limits with an R square value of 0.969, suggesting that the model could be used as a predictor, which converted spectrometric results to HPLC data applicably. The nonzero constant (– 71.45) in the model was not statistically significant (P = 0.394), indicating no significant intercept in the model. Besides, the constant was relatively small, compared to commonly high vegetable nitrate concentrations (> 1,000 *μ*g/g). With a slope of nearly unity and no significant intercept, it is concluded that data derived from both methods are virtually identical.

### 3.4. Results of Spike and Stability Tests

Table 3 shows the results of quality check. The sample solutions that were analyzed on 5 different days within a week resulted in coefficients of variation (CV) lower than 6%, indicating the stability of vegetable samples as well as the precision of the method. The recovery rates calculated from the spike analysis show a range between 74.8 and 91.4%, suggesting that vegetables of different kinds may have different medium abilities to interact with nitrate.

### 3.5. Advantages and Limitations

We have shown that this spectrometric method is reliable and capable of generating data highly correlated to that derived from a standard method using HPLC [17]. In addition, there are several advantages in comparison to other analytic methods (Table 4) [15, 17]. As for expenses, though the ion-exchange SPE cartridges used for this method were not the cheapest, the fee for analysis on spectrometry per sample was the lowest (NT$1,500 ≈ US$49.2), making the total cost per sample analysis the most inexpensive. In terms of time consumption, using a spectrometer for an analytic run took less than a minute, whereas HPLC or IC usually required 10 minutes or longer. Considering more concerns, such as time for stability of instrument and for establishment of calibration, spectrometers are definitely superior to HPLC or IC. Therefore, we conclude that this spectrometric method for leafy vegetable nitrate analysis is quick, accurate, reliable, and cost-effective.

Unlike the HPLC or IC methods that are able to distinguish nitrite from nitrate, this method may not be suitable for samples containing significant levels of nitrite. Fortunately, most leafy vegetables contain nearly no nitrite, evidenced by the previous studies [15, 17] and our HPLC chromatograms showing no peak of nitrite. The only vegetable showing a relatively significant amount of nitrite in the study of Chou et al.'s [17] was broccoli, which appeared to be no fit for this spectrometric method. Vegetables other than leafy ones, such as pumpkins, beans, and carrots, may need testing to assure the availability. Because high contents of nitrate are mostly found from leafy vegetables, with results of the two previous studies and our work we are confident that leafy vegetables are a good fit for analysis using this spectrometric method.

## 4. Conclusions

We confirm that leafy vegetables could be analyzed for nitrate on spectrometry applicably using ion-exchange SPE cartridges as preanalysis treatment to reduce interference from organic matrices. The derived results were close to and highly correlated to that analyzed by a standard HPLC method. In addition, this method is time and money saving with as good accuracy and precision.

## Figures and Tables

**Table 1:** Comparison of mean nitrate concentrations (*μ*g/g) between spectrometric and HPLC analyses of various vegetables.

Vegetable	No. of pairs	Spectrometry	HPLC	Relative Error
		Mean ± SD	Mean ± SD	
Bok coy	3	3464 ± 725	3356 ± 691	3.2%
Cabbage	3	1684 ± 47	1702 ± 30	–1.1%
Celery	3	2896 ± 740	2773 ± 940	4.4%
Chinese cabbage	3	1451 ± 671	1360 ± 485	6.7%
Chinese kale	3	2030 ± 528	2030 ± 613	0.0%
Lettuce	3	1520 ± 615	1324 ± 521	14.9%
Mustard green	3	1455 ± 45	1268 ± 7.3	14.7%
Pak choi	3	3080 ± 299	3013 ± 413	2.2%
Water spinach	3	1839 ± 227	1884 ± 209	–2.4%

**Table 2:** Statistical parameters of linear regression analysis.

Model	Unstandardized Coefficients		Standardized Coefficients	P	95.0% Confidence Interval	
	Value	Standard Error			Lower Bound	Upper Bound
(Constant)	-71.45	82.44		0.394	-241.25	98.35
Slope	0.997	0.036	0.984	<0.001	.923	1.070

**Table 3:** Results from stability tests and spike analyses.

Vegetable	Stability test		Spike analysis	
	N	CV (%)	Concentration without spiking (*μ*g/g)	Recovery rate (%)
Bok coy	5	5.55	1490	74.8
Cabbage	5	5.33	821	75.0
Celery	5	2.40	2287	86.0
Chinese cabbage	5	1.39	1287	84.1
Chinese kale	5	3.26	607	78.0
Lettuce	5	5.49	298	89.0
Mustard green	5	2.40	2469	83.3
Pak choi	5	2.04	3057	91.4
Water spinach	5	4.42	2087	91.4

Mean±SD		3.59±1.63	1600±934	83.7±6.52

CV = coefficient of variation; SD = standard deviation.

**Table 4:** Comparisons among analyses on spectrometry, HPLC and IC.

Method	This method on spectrometry	HPLC ^a^	IC ^b^
Cost of pre-analysis treatment material ^c^	Ion exchange SPE cartridge, NT$243	Syringe filter, NT$26	Ag containing SPE cartridge, NT$388
Cost of instrumental analysis per sample ^d^	NT$1,500	NT$3,000	NT$2,000
Instrumental analysis time per sample	< 1 min	15 min	15 min
Limit of detection	0.1 *μ*g/mL	0.05 *μ*g/mL	< 0.5 *μ*g/mL
Availability	Nitrate only	Nitrite and nitrate	Nitrite and nitrate

^a^ [17]

^b^ [15]

^c^ Monetary values of materials are obtained from local vendors.

^d^ Analysis charges are given by the Institute of Nuclear Energy Research, Atomic Energy Council, Executive Yuan, Taiwan.

## Data Availability

The original data used to support the findings of this study are available from the corresponding author upon request.
